# I’m Very Cautious About Who I Let Into My World: Social Vulnerability for People Living Alone With Dementia

**DOI:** 10.1093/geroni/igaa057.2024

**Published:** 2020-12-16

**Authors:** Kate de Medeiros, Laura Girling

**Affiliations:** 1 Miami University, Oxford, Ohio, United States; 2 University of Maryland, Baltimore County, Baltimore, Maryland, United States

## Abstract

Living alone with Alzheimer’s and related dementias (ADRD) can have many risks including social vulnerability that leads to loneliness. This paper reports findings from 9 people living alone with ADRD who completed in-depth, face-to-face interviews as part of a larger, NIA-sponsored study. Narrative data were analyzed using ATLAS. ti. Thematic findings revealed that although participants received supports (e.g., financial, meal preparation) from others, they lacked opportunities to participate in meaningful engagements with people of their choice (e.g., a friend who lives too far away, a son who is busy). In addition to loneliness resulting from lack of control over their social networks, many also reported that personal changes (e.g., difficulties eating) made them hesitant to seek social engagements. Overall, this paper underscores the need for social programs that extend beyond health-related outcomes and instead speak to subjective wellbeing and social connectivity for this population.

